# Identification of C-Terminal Binding Protein 1 as a Novel NMDA Receptor Interactor

**DOI:** 10.1007/s11064-018-2633-5

**Published:** 2018-10-03

**Authors:** Sarah L. Cousins, F. Anne Stephenson

**Affiliations:** University College London School of Pharmacy, 29/39 Brunswick Square, London, WC1N 1AX UK

**Keywords:** C-terminal binding protein, Ionotropic glutamate receptor, NMDA receptor, Protein–protein interaction

## Abstract

A new *N*-methyl D aspartate neurotransmitter receptor interacting protein has been identified by yeast two-hybrid screening of a mouse brain cDNA library. C-terminal binding protein 1 (CtBP1) was shown to associate with the intracellular C-terminal regions of the *N-*methyl D aspartate receptor subunits GluN2A and GluN2D but not with GluN1-1a cytoplasmic C-terminal region. In yeast mating assays using a series of GluN2A C-terminal truncations, it was demonstrated that the CtBP1 binding domain was localized to GluN2A 1157–1382. The GluN2A binding domain was identified to lie within the CtBP1 161–224 region. CtBP1 co-immunoprecipitated with assembled GluN1/GluN2A receptors expressed in mammalian cells and also, in detergent extracts of adult mouse brain. Co-expression of CtBP1 with GluN1/GluN2A resulted in a significant decrease in receptor cell surface expression. The family of C-terminal binding proteins function primarily as transcriptional co-repressors. However, they are also known to modulate intracellular membrane trafficking mechanisms. Thus the results reported herein describe a putative role for CtBP1 in the regulation of cell surface *N*-methyl D aspartate receptor expression.

## Introduction

Excitatory *N*-methyl-D-aspartate (NMDA) neurotransmitter receptors are key brain proteins because of the central role they play in long term potentiation and long term depression, mechanisms of learning and memory; in synaptogenesis during the development of the central nervous system, and as a potential therapeutic target in neurodegenerative and psychiatric disorders that include stroke, neuropathic pain, epilepsy, schizophrenia, and Alzheimer disease [1, 2]. NMDA receptors are tetramers formed by the co-assembly of two copies of the obligatory GluN1 subunit together with either two copies of a single type of the four GluN2 subunits (i.e. GluN2A to GluN2D), a single copy of two types of the GluN2 subunit class, or a single copy of an GluN2 subunit together with one of the GluN3 class. The NMDA receptor subpopulations have distinct physiological and pharmacological properties, localizations and developmental profiles [3].

At synapses, NMDA receptors are integral components of a macromolecular signalling complex [4]. The membrane associated guanylate kinase (MAGUK) family of scaffolding proteins are key components linking NMDA receptors with intracellular signalling pathways. They bind via their PDZ1 and PDZ domains to a motif, ES(D/E)V that is common to all GluN2 subunit C-termini. However, the four members of the MAGUK family, post-synaptic density 95 (PSD95), chapsyn 110, synapse associated protein-102 (SAP102) and SAP97, have been shown to interact differently with each NMDA receptor subtype [5, 6]. Indeed, an SH3 binding domain within the GluN2A C-terminal tail was identified as a subtype-specific PSD-95 binding site [7]. This differential PSD-95/GluN2A interaction may contribute to the observed differences between GlUN2A and GluN2B with respect to their distribution and lateral mobility [8].

**Fig. 1:**
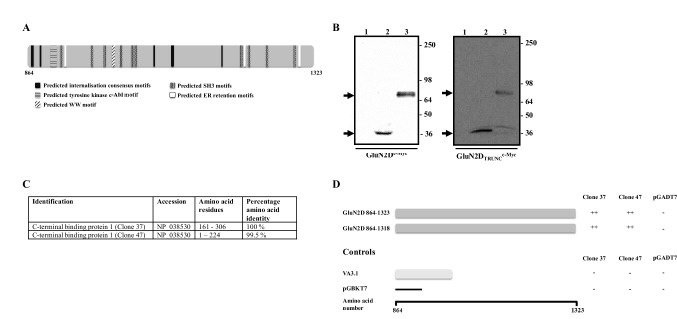
A summary of cDNA library screening strategy and results. **A** Shows the intracellular C-terminal GluNR2D C-terminal domain highlighting putative protein–protein interaction domains. For **B** single colonies of AH109 yeast cells pre-transformed with pGBKT7GluNR2D 864–1323, pGBKT7GluNR2D 864–1318 or pGBKT7 were used to inoculate 5 ml—Trp selection media cultures which were then grown at 30 °C for 48 h at 250 rpm. Proteins were extracted from the resulting yeast cell pellets and analysed by immunoblotting using anti-c-Myc antibodies. The gel layout is the same for both immunoblots where: lane 1 = untransformed AH109 cells; lane 2 = pGBKT7 transformed AH109 cells and lane 3 = pGBKT7GluNR2D 864–1323 (GluN2D^c-Myc^) or pGBKT7GluNR2D 864–1323 (GluN2D^Trunc^) transformed AH109 cells. Arrows denote the positions of GluNR2D 864–1323 and GluNR2D 864–1318. The immunoblots are representative of n = 3 independent transformations. Molecular weight markers × 10^3^ are shown on the right hand side. **C** Is a summary of the characteristics of two interacting clones. **D** A schematic diagram showing the interactions between GluNR2D 864–1323 and GluNR2D 864–1318 and clones 37 and 47

Several reports have suggested that GluN2D-containing receptors are extra-synaptic [9–12]. This together with the findings that GluN2D-containing receptors interacted differently with the MAGUK proteins [5, 6] and the fact that the GluN2D intracellular C-terminal tail contains multiple protein–protein interaction domains (Fig. 1), led to a yeast-two hybrid screen conducted with the aim to identify a GluN2D targeting protein. The screen revealed the identification of a novel, putative NMDA receptor interacting protein, C-terminal binding protein1 (CtBP1). We report these findings in this paper.

## Experimental Procedures

### Constructs and Antibodies

#### Mammalian Expression Constructs

For all NMDA receptor subunit constructs, amino acid numbering begins at the start methionine in the signal peptide. pCISGluN1-C2 and pCISGluN2A were as in [6]. pGW1PSD-95α^c-Myc^ and pCMVneoSAP102c-Myc were kind gifts from Dr M. Sheng (Genentech Inc. USA). The full length mouse CtBP1 variant 1 clone was purchased from Genscript (Piscataway, New Jersey, USA). It was subcloned into pCMV-4a, using *Eco*RI at the 5′ and *Hin*dIII 3′ end using primers 5′AAAA GAATTC ATGGGCAGCTCCCACTTG3′ and 5′AAAA AAGCTT CAACTGGTCACTCGTATG3′ which generated when expressed, CtBP1 with a FLAG tag at the N-terminus.

#### Yeast Two-Hybrid Expression Constructs

The DNAs encoding the C-terminal domain constructs GluN2D 864–1323 and GluN2D 864–1318 were generated by PCR from the appropriate mammalian expression construct and subcloned in frame into the *Bam*HI/*Eco*RI sites of the pGBKT7 yeast bait vector to generate pGBKT7GluND and pGBT7GluN2D^Trunc^, i.e. the GluN2D C-terminal domain lacking the ESEV, PSD-95 membrane associated guanylate kinase (MAGUK) binding domain. The C-terminal constructs pGBKT7GluN1-1a 834–938; pGBKT7GluN2A (encoding GluN2A 838–1464); pGBKT7GluN2A^1460^ (i.e. pGBKT7GluN2A^Trunc^); pGBKT7GluN2A^1441^; pGBKT7GluN2A^1420^; pGBKT7GluN2A^1420-ASDA^; pGBKT7GluN2A^1382^ and pGBKT7GluN2A^1157^ were generated as in [7]. Expression of all constructs was verified by immunoblotting ([7] and Fig. 1).

#### Antibodies

Affinity-purified anti-GluN1 C2 (911–920); anti-GluN2A (44–58 Cys) and anti-GluN2A (1381–1394) antibodies were raised and characterized as previously reported [6]. Mouse monoclonal anti-FLAG M2 antibodies were from Sigma-Aldrich (Poole, UK) and anti- c-Myc Clone 4A6 mouse monoclonal antibodies from Upstate USA Inc. (Charlottesville, VA, USA). Anti-CtBP1 antibodies raised in mice were from Sigma-Aldrich (Poole, UK) where the antigen used was a glutathione S-transferase full length (i.e. 1–429) CtBP1 fusion protein.

#### Yeast Two-Hybrid Screening

The mouse brain cDNA library was sourced from 200 BALB/c males, aged 9–12 weeks, normal whole brains. The number of independent clones present was 3.5 × 10^6^ which had an average insert size of 2.0 kb and a range of 0.4–4.0 kb. The library was amplified as instructed by the manufacturer (Clontech, a TakaraBio company, St-Germain-en-Laye, France). The library was amplified so that each independent clone was represented at least three times thereby ensuring that rare clones were present. The number of independent clones in the amplified library was 10.5 × 10^6^. For the library screen, the method of [13] was followed. Pre-transformed with pGBKT7GluN2D 864–1323, AH109 *S. cerevisiae* yeast cells were co-transformed with cDNA mouse brain library plasmid DNA and cultured on -Leu/-Trp/-His/-Ade selective dropout 2% (w/v) agar plates for 5–7 days at 30 °C. Resulting colonies were streaked onto fresh -Leu/-Trp/-His/-Ade plates every 4 days for a total of three times. Plasmid DNAs from colonies surviving this rigorous screen, i.e. encoding putative positive GluN2D 864–1323 interactors, were isolated and characterised by yeast mating assays as below.

#### Yeast Two-Hybrid Interaction Mating Assays

Yeast two-hybrid interaction mating assays to verify protein–protein interactions were carried out for either pGBKT7GluN2D 864–1323; pGBKT7GluN2D 864–1318; pGBKT7GluN1-1a 834–938; pGBKT7GluN2A (encoding GluN2A 838–1464); pGBKT7GluN2A 838–1460 (i.e. pGBKT7GluN2A^Trunc^); pGBKT7GluN2A 838–1441; pGBKT7GluN2A 838–1420; pGBKT7GluN2A 838–1420-ASDA; pGBKT7GluN2A 838–1382; pGBKT7GluN2A 838–1157 (bait constructs) and, pGADT7CtBP1 1–224 and pGADT7CtBP1 161–306 (fish constructs) exactly as described in [7]. Positive and negative controls were always carried out in parallel. The positive control was AH109 S. cerevisiae cells pre-transformed with pTD1-1 and Y187 S. cerevisiae cells pre-transformed with pVA3-1. The negative controls were either empty pGBKT7- and pGADT7- vectors or empty fish or bait respectively to check for auto-activation of either the bait or fish constructs.

#### Mammalian Cell Transfections

Human embryonic kidney (HEK) 293 cells were cultured and transfected using the calcium phosphate method as previously described [6]. Cells were incubated post-transfection in the presence of 1 mM ketamine to prevent NMDA receptor-mediated cytotoxicity [6]. HEK 293 cells were transfected in parallel with either CtBP1^FLAG^ alone (10 µg) or, GluN1-C2 + GluN2A + either CtBP1, PSD-95 or SAP102 clones. The ratio of DNAs for the transfections were GluN1: GluN2: CtBP1, PSD-95 or SAP102, 1:3:4 which corresponded to 2.5 µg:7.5 µg:10 µg for a 20 µg total DNA for transfection of a 1 × 250 ml flask. For transfections where cell surface NMDA receptor expression was measured, HEK 293 cells were subcultured overnight prior to transfection in poly-d-lysine (100 µg/ml)-coated 24-well dishes and 0.5 µg total plasmid DNA was used per well.

#### Immunoprecipitation Assays

HEK 293 cells were harvested 24 h post-transfection, cell homogenates prepared and solubilised for 1 h at 4 °C at a concentration of 1.5 mg protein/ml with solubilisation buffer (50 mM Tris-citrate, pH 7.4, 240 mM NaCl, 5 mM EDTA, 5 mM EGTA, 1% (v/v) Triton X-100 containing benzamidine (1 µg/ml), bacitracin (1 µg/ml), soybean trypsin inhibitor (1 µg/ml), chicken egg trypsin inhibitor (1 µg/ml) and phenylmethylsulphonyl fluoride (1 mM)). Samples were diluted to 1 mg protein/ml with the above solubilisation buffer and solubilised material was collected by centrifugation at 100,000×g for 40 min at 4 °C. The detergent extracts were incubated with affinity-purified rabbit anti-GluN1 C2 antibodies (5 µg) or protein A purified non-immune rabbit Ig (5 µg) as control overnight at 4 °C. Protein A Sepharose (25 µl) was added and samples incubated for 1 h at 4 °C. Immune pellets were collected by centrifugation for 15 s at 600×g, washed with 3 × 1 ml solubilisation buffer and then analyzed by immunoblotting.

For immunoprecipitations from native tissue, the P2 membrane fraction was made from adult rat brain minus the cerebellum and 1% (w/v) sodium deoxycholate detergent solubilised 100,000×g extracts were prepared as described [14]. Samples were incubated with anti-GluN1 C2 or non-immune rabbit Ig primary antibodies for 16 h at 4 °C. Immunoprecipitations were completed as above.

#### Immunoblottting

Immunoblotting was performed as previously described using 25–50 µg protein/sample precipitated using the chloroform/methanol method and SDS-PAGE under reducing conditions in 7.5% (w/v) polyacrylamide slab minigels all as previously described [5]. Primary antibodies used were: anti-GluN1 C2 (911–920), anti-GluN2A (1381–1394) or anti-FLAG M2 primary antibodies. Rabbit or mouse horseradish-linked secondary antibodies (GE Healthcare, Chalfont St Giles, Bucks., UK) were used at a final dilution of 1:2000 and immunoreactivities were detected using the ECL western blotting system.

#### Determination of NMDA Receptor Cell Surface Expression by ELISA

NMDA receptor cell surface expression was carried out by ELISA assay using affinity-purified antibodies directed against an extracellular epitope of GluN2A i.e. anti-GluNR2A 44–58 Cys at a concentration of 0.125 µg/ml all exactly as described in [5].

## Results and Discussion

### cDNA Library Screening: Identification of a Putative NMDA GluN2D Interacting Protein

**Fig. 2:**
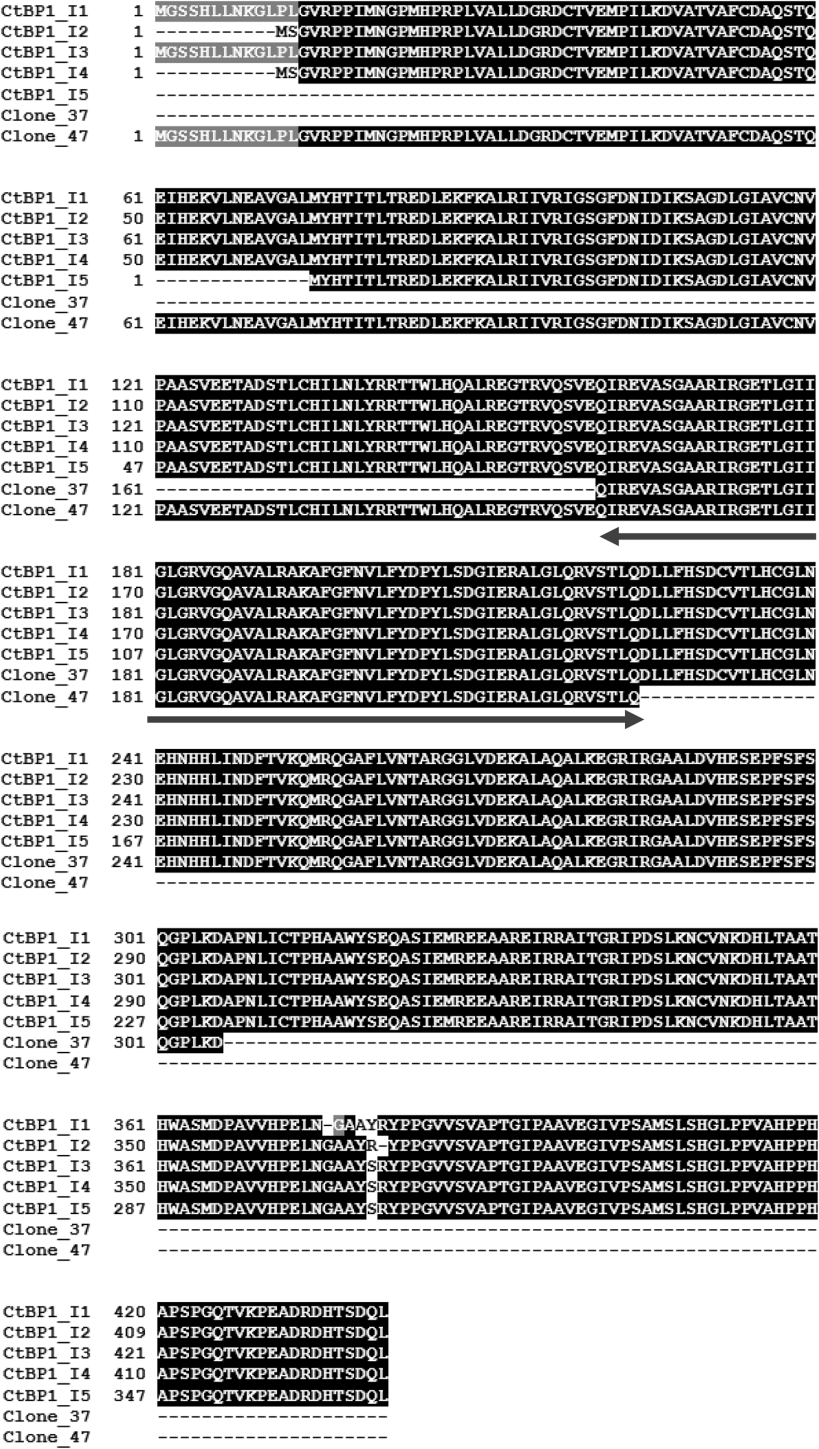
An alignment of CtBP1 and NR2D C-terminal domain interacting clones, clone 37 and clone 47. An alignment of the five known isoforms of CtBP1 with putative interacting clones, 37 and 47. The arrow denotes the overlapping region shared by clone 37 and clone 47

The screen of the mouse brain cDNA library with the bait, pGBKT7GluN2D 864–1323, yielded 240 positive interactors. From these, 60 strong and 60 weak interactors were randomly selected and each were subjected to yeast mating assays. From the selected 120 clones, 20 (19 from the pool of strong interactors and 1 from the pool of weak interactors) were real interactors in the yeast mating assays. For these 20 clones, each colony was analysed in triplicate to determine the presence of a cDNA insert, the size of the insert and if the insert was in frame with the activation domain. Ten clones satisfied these criteria. These were subsequently DNA sequenced. Within these ten, six clones were eliminated as they are known false positives leaving four final clones. It was found that clones 37 and 47 encoded the same protein and overlapped in the DNA sequence obtained. Database searches showed that they encoded CtBP1 161–306 (clone 37) and CtBP1 1–224 (clone 47) (Fig. 1). Notably, deletion of the GluN2D ESDV PSD-95 C-terminal binding domain did not affect the interaction with clones 37 and 47 (Fig. 1d). There are five known isoforms of CtBP1 named CtBP1-I1-I5. Figure 2 shows the alignment of clones 37 and 47 with the five CtBP1 isoforms. Clone 47 shares amino acid sequence identity with CtBP1-I1 and CtBP1-I3 whereas clone 37 shares amino acid sequence identity with all five CtBP1 isoforms. The CtBP1 amino acid sequence that is common to both clones, i.e. CtBP1 161–224 is identical across all five CtBP1 variants (Fig. 2).

### CtBP1 1–224 and CtBP1 161–306 Associate with GluN2A: Refinement of GluN2A/CtBP1 Interaction Site

**Fig. 3:**
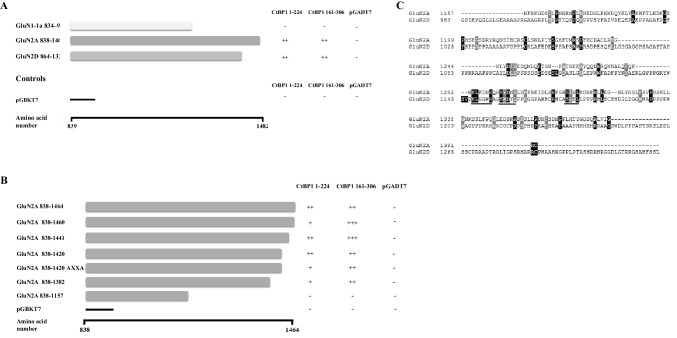
Identification of the GluN2A amino acid sequence that mediates association with CtBP1 1–224 and C-tBP1 protein 161–306. **A** and **B** Y189 *S. cerevisiae* yeast cells transformed with C-terminal bait constructs, pGBKT7GluN1-1a 834–938, pGBKT7GluNR2A, pGBKT7GluNR2D 864–1323 or truncations, as shown of GluNR2A C-terminal bait constructs as shown and AH109 S. cerevisiae cells transformed with pGADT7CtBP1 1–224 or pGADT7CtBP1 161–306 were mated and grown on -Leu/-Trp/-His/-Ade agar plates, and the number of diploid colonies counted after 7 days of incubation at 30 °C all as described under “Experimental Procedures”. The figures depict the bait and fish constructs and the resulting diploid colonies.. The number of diploid colonies was semi-quantified by the number of colonies thus +++ = 500 +; ++ = 250–499; + = 1–241 and − = 0 colonies. **C** A schematic diagram highlighting possible protein–protein interaction motifs shared between GluNR2A and GluNR2D

The interaction between CtBP1 and NMDA receptor subunits merited further study since CtBP1 1–224 and CtBP1 161–306 were identified in a rigorous yeast two hybrid cDNA screen and importantly were overlapping with respect to the amino acid sequences they encoded. In the first instance, the specificity of CtBP1/NMDA receptor subunits was investigated using yeast mating assays with appropriate positive and negative controls. Firstly, it was found that both CtBP1 1–224 and CtBP1 161–306 both interacted with the C-terminal tail of GluN2A, i.e. GluN2A 838–1464, with a qualitatively similar affinity to GluN2D 864–1323 (Fig. 3). It was found that CtBP1 did not associate with the GuN1-1a C-terminal domain (Fig. 3).

Refinement of the CtBP1 binding site on GluN2A was determined using a series of GluN2A C-terminal deletion constructs. These GluN2A constructs were designed for previous studies in which a second GluN2A PSD-95 binding site was identified [7]. Truncation of GluN2A at amino acid 1157 resulted in the loss of interaction between GluN2A and both CtBP1 1–224 and CtBP1 161–306. Although there were some qualitative differences in the strength of the association of GluN2A with CtBP1 1–224 and GluN2A with CtBP1 161–306, both showed similar profiles with the CtBP1 GluN2A binding site identified as lying between GluN2A 1157–1382 (Fig. 3). This region does not include the non-ESDV Src homology three domain-binding motif 1382–1389 [7]. Since the original cDNA library screen identified CtBP1 as a GluN2D interacting protein, the intracellular, C-terminal amino acid sequences of GluN2A and GluN2D were compared aiming to identify common motifs. Figure 3c shows an alignment of the two sequences. Three conserved amino acid motifs are found between GluN2A and GluN2D i.e. KLXXXR; SXDX(I/L) and S(I/L)XL (Fig. 3c).

### CtBP1 Co-immunoprecipitates with Assembled GluN1/GluN2A NMDA Receptors from Detergent Extracts of Receptors Expressed in HEK 293 Cells of Native Brain Tissue

**Fig. 4:**
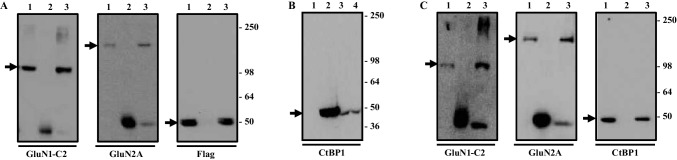
CtBP1 co-immunoprecipitates with GluN2A expressed in HEK 293 cells and from native brain tissue. **A** and **B** HEK 293 cells were either co-transfected with GluN1-1a/GluN2A/CtBP1^FLAG^ clones or pCIS or the CtBP1^FLAG^ clones alone, cells cultured for 48 h, 100,000×g detergent extracts (**A**) or cell homogenates (**B**) were prepared and immunoprecipitations (**A**) or immunoblotting (**B**) carried out all as described in “Experimental Procedures”. Immunoprecipitations were carried out using with anti-GluN1 C2 or non-immune Ig antibodies and immunoblots were probed with anti-GluN1 C2, anti-GluNR2A (1381–1394) and anti-FLAG antibodies as shown. The results are representative of n = 3 independent transfections. **B** Immunoblots of transfected cell homogenates were probed with anti-CtBP antibodies. **C** Immunoprecipitations from 100,000×g detergent extracts of whole rat brain were carried out using anti-GluN1 C2 or non-immune Ig antibodies and immunoblots were probed with the antibodies as shown in the abscissa. The immunoblots are representative of n = 3 independent immunoprecipitations. For **A** and **B** the gel layout is the same where lane 1 = input; lane 2 = non-immune Ig pellet; lane 3 = anti = GluR1 C2 immune pellet. For C, lane 1 = HEK 293 cells transfected with pCIS; lanes 2–4, homogenates of HEK 293 cells transfected with the CtBP1^FLAG^ clone where lane 2 = µg protein; lane 3 = µg protein and lane 4 = µg protein Molecular weight markers × 10^3^ are shown on the right hand side

To determine if full length CtBP1 co-associates with NMDA receptor complexes, FLAG-tagged full length CtBP1-I1 was co-expressed with GluN1/GluN2A in HEK 293 cells and immunoprecipitations were carried out using anti-GluN1 C2 antibodies or non-immune Ig as a control. Anti-GluN1 antibodies were selected for immunoprecipitations since CtBP1 does not associate with GluN1 (Fig. 3). Therefore, if anti-CtBP1 immunoreactivity was evident in immune pellets, it would demonstrate that CtBP1 associated with assembled GluN1/GluN2A NMDA receptors. The results of immunoblots of immune pellets are shown in Fig. 4. A band of M_r_ ~ 120 kDa was evident in anti-GluN1 C2 immune pellets but not in non-immune Ig controls demonstrating successful immunoprecipitation. A band with M_r_ ~ 180 kDa was detected in anti-GluN1 C2 but not non-immune Ig pellets indicating immunoprecipitation of assembled GluN1/GluN2A NMDA receptors. Anti-FLAG immunoreactivity, M_r_ ~ 50 kDa was also detected in anti-GluN1 C2 but not control immune pellets thus demonstrating that full length CtBP1-I1 does indeed co-immunoprecipitate with assembled GluN1/GluN2A NMDA receptors (Fig. 4).

Immunoprecipitations were also carried out from detergent extracts of adult rat brain. As for heterologous co-expression of GluN1/GluN2A/CtBP1-I1, anti-GluN1 C2 primary antibodies specifically immunoprecipitated GluN1 and GluN2A immunoreactive bands (Fig. 4c). Further, anti-CtBP1 immunoreactivity was present in immune but not non-immune pellets. Immunoprecipitation with only anti-GluN1 antibodies does not distinguish between GluN1/CtBP1 and GluN1/GluN2A/CtBP1 immunoprecipitated complexes. We cannot exclude the possibility that although CtBP1 does not interact directly with GluN1-1a in yeast mating assays, it may associate and thus co-immunoprecipitate with GluN1 from brain detergent extracts via an intermediary, brain protein. However, given the yeast mating assay findings that CtBP1 interacts directly with GluN2A but not GluN1-1a, this seems unlikely. Thus it is a reasonable conclusion that in the brain and in heterologous expression systems, CtBP1 co-immunoprecipitates with native, assembled GluN1/GluN2A NMDA receptors.

### Co-expression of CtBP1 Results in a Decrease of GluN1/GluN2A Cell Surface Expression

**Fig. 5:**
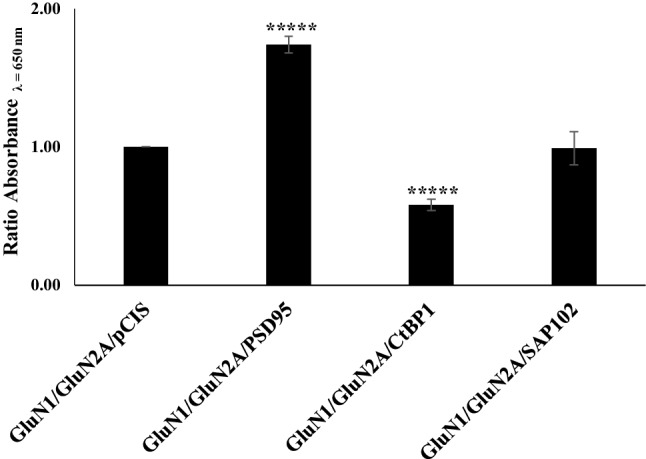
Co-expression of CtBP1 with GluN1/GluN2A results in decreased cell surface expression. HEK 293 cells were co-transfected with either GluNR1-C2 + GluN2A + either CtBP1, PSD-95 or SAP102 clones and cell surface expressed NMDA receptors measured by ELISA assay using either anti-NR2A 44–58 Cys affinity-purified antibodies. The results are expressed as the ratio of absorbance in the presence to the absence of respective PSD-95, SAP102 or CtBP. The results are the mean ± SEM of triplicate samples from n = 7 independent transfections experiments. *****p < 0.0001; ****p < 0.005

There are four members of the CtBP family, (CtBP1, CtBP2, CtBP3/BARS and RIBEYE). The homologous proteins were originally identified as binding partners for the E1A-transforming proteins and are now recognized to function primarily as transcriptional co-repressors. CtBP1 and CtBP2 are both widely expressed in mammalian tissues including the brain (reviewed in [15, 16]). With respect to their subcellular distribution, CtBP2 has a nuclear localization signal and accumulates within the nucleus whereas CtBP1 is found in both the cytoplasm and in the nucleus. In addition to the repressor function the family of CtBP proteins have been shown to have different cellular functions in Golgi membranes, and in synaptic ribbons [17]. Since CtBP1 has been implicated in mechanisms of intracellular trafficking [18], thus it was of interest to determine what effect CtBP1 had on the cell surface expression of NMDA receptors. GluN1/GluN2A NMDA receptors were co-expressed transiently in HEK 293 cells and cell surface expression measured by an ELISA using antibodies directed against the GluN2A extracellular domain. In parallel, GluN1/GluN2A were expressed with PSD-95 or SAP102 as positive and negative controls. The results are shown in Fig. 5. As previously published co-expression with PSD-95 resulted in an enhanced GluN1/GluN2A surface expression of 175% ± 6 (n = 7), (p < 0.0001) whereas co-expression with SAP102 had no significant effect of receptor surface expression [5]. The values for SAP102 co-expression were: 99% ± 0.12 (n = 7), (p < 0.1). Co-expression with CtBP1 resulted in a decrease in GluN1/GluN2A surface expression, i.e. 58% ± 4 (n = 7), (p < 0.0001) with respect to control (Fig. 5).

## Concluding Remarks

In summary, a novel protein–protein interaction between CtBP1 and GluN2A-containing NMDA receptors has been discovered. We have refined the sites of association between the two proteins. Further, we have shown that CtBP1 co-immunoprecipitates with assembled GluN1/GluN2A receptors and importantly, expression of CtBP1 with GluN1/GluN2A resulted in a decreased cell surface receptor expression. Co-immunoprecipitation does not show that two proteins interact directly but since the association of CtBP1 with NMDA receptor NR2 subunits was found by yeast two-hybrid interactions, this implies that the link between the two proteins is direct. Optimally however, an additional method such as pull down assays needs to be carried out to substantiate direct association between CtBP and NMDA receptors. Further, co-distribution studies are also essential. The original yeast two-hybrid screen indicated that CtBP1 associates with GluN2D-containing NMDA receptors. Preliminary yeast mating assays findings revealed that this is also the case for GluN2B, i.e. C-terminal GluN2B domains interact with CtBP1 1–224 and CtBP1 161–306. Further studies are required to determine if CtBP1 is universal with regard to NMDA receptor subtype association and regulation.

The primary function of CtBP1 is as a repressor of transcription but as described above, it is also implicated in intracellular trafficking mechanisms. In neuronal cells CtBP1 has a dual localization. It is found in the nucleus and importantly, it is enriched in synaptosomal fractions [19]. It may be speculated that CtBPs are involved in synapse to nuclear signalling. Intriguingly however, CtBP proteins were shown to co-immunoprecipitate from brain extracts with neuronal nitric oxide synthase, a known downstream signalling pathway activated by glutamate and NMDA receptors [20]. CtBP expressed in Madin–Darby canine kidney cells has a primarily nuclear localization [20]. But when neuronal nitric oxide synthase is co-expressed with CtBP, this resulted in a shift to a more cytosolic localization [20]. Further, there are examples of altered in CtBP expression in neurodegenerative conditions in which NMDA receptor dysfunction is implicated (reviewed in [16]). Alternatively, it may just control membrane trafficking of receptors. Future studies will yield further insights into the functional significance of NMDA receptor/CtBP1 interactions.

## Abbreviations

Ade: Adenine
CtBP: C-terminal binding protein
Leu: Leucine
His: Histidine
MAGUK: Membrane associated guanylate kinase
NMDA: *N*-methyl-D-aspartate
PSD: Post-synaptic density
SAP: Synapse associated protein
